# Pancreatic islet transplantation: current advances and challenges

**DOI:** 10.3389/fimmu.2024.1391504

**Published:** 2024-06-03

**Authors:** Qi Wang, Yu-xi Huang, Long Liu, Xiao-hong Zhao, Yi Sun, Xinli Mao, Shao-wei Li

**Affiliations:** ^1^ Department of Hepatobiliary and Pancreatic Surgery, Taizhou Hospital of Zhejiang Province Affiliated to Wenzhou Medical University, Linhai, Zhejiang, China; ^2^ Department of Hepatobiliary and Pancreatic Surgery, Second Affiliated Hospital, School of Medicine, Zhejiang University, Hangzhou, China; ^3^ Department of Pharmacy, Taizhou Hospital, Zhejiang University, Taizhou, Zhejiang, China; ^4^ MRL Global Medical Affairs, MSD China, Shanghai, China; ^5^ Department of Gastroenterology, Taizhou Hospital of Zhejiang Province Affiliated to Wenzhou Medical University, Linhai, Zhejiang, China; ^6^ Key Laboratory of Minimally Invasive Techniques and Rapid Rehabilitation of Digestive System Tumor of Zhejiang Province, Taizhou Hospital Affiliated to Wenzhou Medical University, Linhai, Zhejiang, China

**Keywords:** pancreatic islet, transplantation, long-term outcomes, MSC/Treg, co-transplantation

## Abstract

Diabetes is a prevalent chronic disease that traditionally requires severe reliance on medication for treatment. Oral medication and exogenous insulin can only temporarily maintain blood glucose levels and do not cure the disease. Most patients need life-long injections of exogenous insulin. In recent years, advances in islet transplantation have significantly advanced the treatment of diabetes, allowing patients to discontinue exogenous insulin and avoid complications.Long-term follow-up results from recent reports on islet transplantation suggest that they provide significant therapeutic benefit although patients still require immunotherapy, suggesting the importance of future transplantation strategies. Although organ shortage remains the primary obstacle for the development of islet transplantation, new sources of islet cells, such as stem cells and porcine islet cells, have been proposed, and are gradually being incorporated into clinical research. Further research on new transplantation sites, such as the subcutaneous space and mesenteric fat, may eventually replace the traditional portal vein intra-islet cell infusion. Additionally, the immunological rejection reaction in islet transplantation will be resolved through the combined application of immunosuppressant agents, islet encapsulation technology, and the most promising mesenchymal stem cells/regulatory T cell and islet cell combined transplantation cell therapy. This review summarizes the progress achieved in islet transplantation, and discusses the research progress and potential solutions to the challenges faced.

## 1 Introduction

Type 1 diabetes (T1D) is a chronic progressive metabolic disorder characterized by hyperglycemia due to destruction of pancreatic β-cells leading to severe insulin deficiency (1). In the early stages, blood sugar levels can be controlled within the normal range using oral hypoglycemic drugs or insulin injections. However, for some patients with advanced diabetes, these interventions are limited in effectiveness and cannot prevent complications, such as metabolic disorders, vascular diseases, and nerve damage. Severe cases can lead to limb necrosis, blindness, kidney failure, and life-threatening conditions (2–4). Although significant progress has been made in diabetes treatment in recent years with new technologies and medications, such as insulin pumps and continuous glucose monitoring devices, the treatment of diabetes remains a significant burden for patients because of the need for dynamic blood sugar monitoring and adjustment. Therefore, searching for new treatment methods is a major issue in the field of diabetes.

Pancreatic islet transplantation (IT) is a procedure that involves the purification of pancreatic islet cells from a donor pancreas, whether it is xenogeneic and their infusion into the patient’s body, mainly through the portal vein. This establishes an endogenous glucose-dependent insulin secretion system, restoring physiological insulin secretion patterns and achieving real-time, accurate blood glucose control. In the long term, it can improve diabetic complications and enable insulin independence, ultimately aiming to cure diabetes. It is considered an ideal solution for diabetes (5). IT has garnered widespread attention as an effective treatment for diabetes. However, many difficulties and challenges have hindered its development (6).

Organ shortage is a global issue and hampers the development of pancreatic IT. Approximately 8,000 organ donations occur annually, but less than one-third of the pancreatic organs are usable for IT (7, 8). The long-term clinical prognosis of patients undergoing traditional portal vein transplantation is poor. Studies have shown that post-transplantation inflammatory and immune rejection reactions can lead to up to 60% pancreatic islet dysfunction or necrosis. Furthermore, complications such as portal hypertension, bleeding, and thrombosis can occur during the portal vein transplantation procedure (9).

In response to these issues, numerous researchers have proposed solutions, and the main research directions to address the shortage of pancreatic islet organs focus on stem cell-derived and porcine-derived islet cells. In terms of selecting new transplant sites, options such as a subcutaneous pocket and the greater omentum have certain advantages compared to the traditional portal vein injection method. In addition, islet encapsulation technology and cellular therapy for combined transplantation of MSC/Treg and islet cells are also under active development to induce immune tolerance in transplant recipients.

We herein report an overview of the current long-term prognosis of patients following IT. Then, we discuss and elaborate on the challenges faced in the IT process and the recent progress of the corresponding solutions. We hope that this information will offer guidance and reference for further research in the field of IT.

## 2 Current outcomes of pancreatic islet transplantation

Clinical IT has been carried out since the 1970s (10), however, for various reasons, its clinical efficacy has not been satisfactory. It was not until 2000 that Shapiro et al. (11) proposed and established a set of standards, including donor selection, transplantation of islet equivalents, and postoperative immunosuppressive regimens. They used a large number of isolated islet cells for transplantation and implemented a new protocol after surgery using a corticosteroid-free regimen and reduced doses of calcium channel blockers (sirolimus, low-dose tacrolimus, and daclizumab), known as the “Edmonton protocol” (11). Once this protocol was promoted, clinical results showed significant improvement, marking an important milestone in clinical IT. In 2006, a clinical islet transplantation trial using the Edmonton protocol (NCT00014911) was published, in which 36 subjects with T1D were enrolled at nine transplant centers for islet transplantation using the Edmonton protocol, with insulin independence and good glycemic control as the endpoint 1 year after transplantation. Results showed that a total of 16 subjects met the primary endpoint, including 5 subjects who remained insulin independent 2 years after transplantation (12). This clinical trial suggests that islet transplantation using the Edmonton protocol can restore long-term endogenous insulin production and stabilize blood glucose levels in T1D patients, but insulin independence may not persist. It may be necessary to continue improving the immunosuppressive regimen to achieve longer insulin independence after islet transplantation. We summarize some clinical trials of immunosuppressive regimen (**Table 1**) and using porcine islets in non-human primates (**Table 2**).

**Table 1:** Different immunosuppressive regimens in islet transplantation.

Immunosuppression therapy	Result	References
Sirolimus, tacrolimus, and daclizumab	Achieved sustained insulin independence for 11.9 months	(11, 13)
Sirolimus or mycophenolate, belatacept (BELA) or efalizumab (EFA)	Achieving insulin independence after one or two islet transplants	(14)
Thymoglobulin and sirolimus, efalizumab, mycophenolic acid (MMF)	All patients achieved insulin independence and complete remission of hypoglycemic episodes after the last islet transplant	(15)
Anti-CD3 mAb and sirolimus, maintained with sirolimus and reduced-dose tacrolimus	Four of six recipients achieved and maintained insulin independence with an increased percentage of CD4+ T cells	(16)
Antithymocyte globulin (ATG), daclizumab, and etanercept, maintained with mycophenolate mofetil, sirolimus, and no or low-dose tacrolimus	Insulin independence and absence of hypoglycemia was achieved in all 8 recipients	(17)
Daclizumab, sirolimus, tacrolimus, etanercept, exenatide	Improves islet graft function and contributes to insulin independence with reduced islets	(18)
Thymoglobulin induction, and doubleblockage of IL-1β and TNF-α as well as sirolimus-free immunosuppression	Only one islet infusion is required, significantly improving the efficacy of clinical islet transplantation	(19)
Rapamycin, ATG, steroids and interleukin-1Ra, rapamycin, mycophenolate mofetil treatment as maintenance therapy	This regimen is feasible and safe but less efficient in maintaining graft survival than other regimens based on T-cell depletion	(20)
Induction immunosuppression with T cell depletion and/or TNF-α inhibition; and maintenance with both mechanistic target of rapamycin (mTOR) and calcineurin inhibitors	Safe to use and exerts a great and significant benefit in blood glucose control	(21)
Alemtuzumab, basiliximab, maintained withtacrolimus, mycophenolatemofetil, and prednisolone	This protocol for postrenal islet transplantation significantly improves islet allograft function and improves glycemic control	(22)

**Table 2:** Immunosuppressive protocol for transplantation of porcine pancreatic islets into nonhuman primates.

Immunosuppressive drugs	Graft survival time	References
Anti‐CD154 mAb, basiliximab, belatacept, sirolimus	>140 days	(23)
CD154-specific and CD25-specific mAb, FTY720 (or tacrolimus), everolimus and leflunomide	>100 days	(24)
CD40-specific monoclonal antibody (Chi220), basiliximab, belatacept, sirolimus	203 days	(25)
Belatacept and mycophenolate, LFA-1 blockade, basiliximab, tacrolimus,	111 days	(26)
Cobra venom factor (CVF), anti-CD154 mAb, low-dose Sirolimus, anti-thymocyte globulin (ATG),Tregs	603 days	(27)
ATG, anti‐CD40 mAb, CVF, adalimumab, sirolimus, with or without belatacept or tacrolimus	60 days	(28)

**Figure 1:**
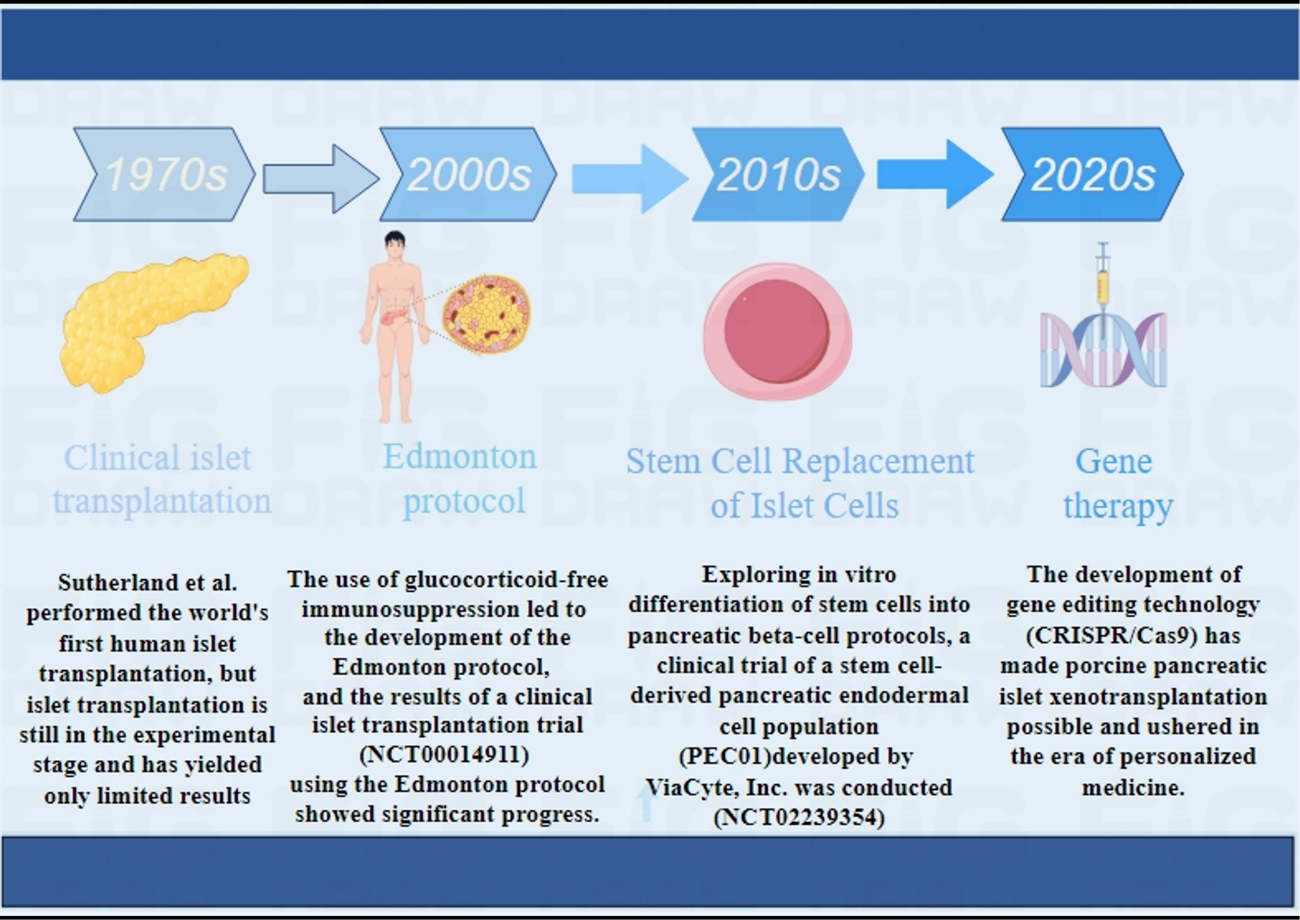
Main development process of clinical islet transplantation.1970S (10), 2000S (11, 12), 2010S (29, 30), 2020S (31, 32).

In recent years, several research teams have published studies on the long-term progress of IT, affirming its therapeutic effects and providing new ideas for future treatment protocols (**Figure 1**). In 2016, Bernhard et al. published a phase III clinical trial for the treatment of severe hypoglycemic complications in T1DM patients through IT. The trial was conducted at 8 centers in North America and included 48 T1DM patients who had been suffering for over 5 years. During the trial, each patient underwent one or more ITs. The primary endpoints of the trial were achieving HbA1c <7.0% (53 mmol/mol) within the first year after the first transplant and avoiding severe hypoglycemic events (SHEs) from day 28 to day 365. The results showed that 87.5% of the participants successfully reached the primary endpoints within one year. IT enables blood sugar control for patients with refractory SHEs and should be considered when other treatments are ineffective (33). In 2023, the team conducted a follow-up investigation of 398 patients with T1DM and SHEs registered in the Collaborative Islet Transplant Registry (CITR). They identified 4 factors that are most beneficial for IT: patients ≥35 years old, infusion of 325,000 islet equivalents, immunosuppression with T cell depletion or TNF-α inhibition, and the use of rapamycin (mTOR) and calcineurin inhibitors. When islet transplant recipients reach the milestone of 5 years after their last islet cell infusion, approximately 95% of patients who meet these 4 common factors experience no SHEs and greatly benefit from improved glycemic control (13).

In 2022, Marfil-Garza et al. from the University of Alberta, Edmonton, Canada, published a study on the long-term results of pancreatic islet cell transplantation over a period of 20 years. This is the largest cohort study to date on the long-term outcomes of IT, including 255 patients from the Edmonton Protocol. This study showed that despite the need for chronic immunosuppression therapy, islet cell transplantation demonstrated good long-term safety. In this study, the median follow-up time was 7.4 years, with 90% patient survival and a median graft survival of 5.9 years. Patients surviving post-transplant exhibit better insulin sensitivity and more stable blood glucose control than non-survivors (34). This study is significant for understanding the long-term effects of islet cell transplantation and for identifying predictive factors. However, further research is needed to validate these results and to continue to evaluate the risks and benefits of IT for better treatment choices for patients.

In a retrospective, multicenter, observational cohort study, 1210 patients from the Pancreatic Islet Transplantation Collaborative Registry at 39 centers worldwide were included. The study demonstrated a linear inverse relationship between primary graft function (PGF) at one month post-most recent IT and the five-year cumulative incidence of adverse outcomes. This suggests an association between early transplantation potential and long-term clinical significance, which has important implications for β-cell replacement therapies. Anticipated clinical outcomes can guide personalized decisions regarding repeat islet injections based on a predefined islet quality threshold, informing current practice. In future trials, PGF may serve as an early and reliable surrogate endpoint for successful IT. These findings highlight the potential of evaluating and optimizing early IT to improve current β-cell replacement outcomes through an enhanced islet survival and function post-transplant. This can enhance the effectiveness of IT and improve patient prognoses (35).

In conclusion, the latest research and clinical data unequivocally support the safety and efficacy of pancreatic islet cell transplantation in T1DM treatment. Furthermore, these studies offer promising new directions for further optimization of IT and for achieving long-term success.

## 3 β-cell replacement options: stem cells and porcine islets

Pancreatic IT holds great promise in the treatment of T1DM. However, the scarcity of pancreatic islets limits the development of this technique. Several research teams have proposed different solutions. Currently, the main focus of pancreatic cell replacement strategies is on stem cells, including embryonic stem cells (ESCs) and induced pluripotent stem cells (iPSCs), as well as porcine islets.

### 3.1 ESCs/iPSCs differentiate into islet β-cells

**Figure 2:**
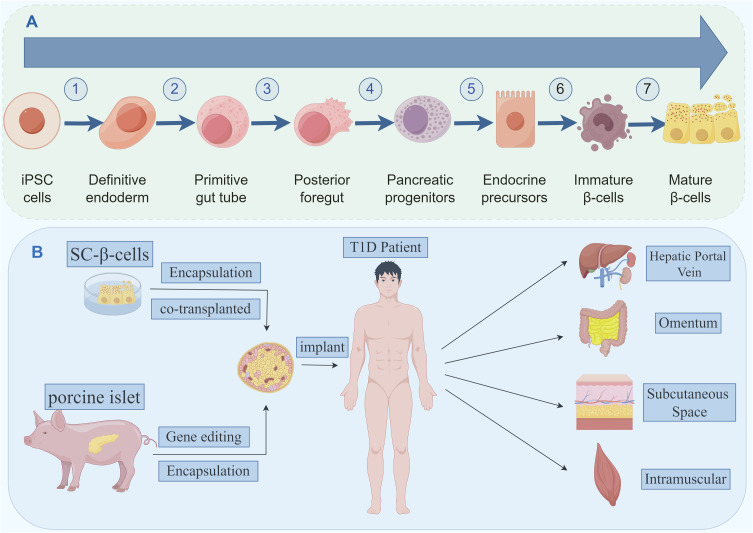
**(A)** The seven stages of differentiation of iPSCs into mature β-like cells. **(B)** Stem cell-derived beta cells and porcine islet-derived beta cells, which can be modified by a number of techniques and transplanted to potential transplantation sites.

The strategy for *in vitro* differentiation of ESCs/iPSCs into pancreatic cells mimics the molecular regulatory mechanisms of pancreatic development *in vivo*. It involves the use of a combination of growth factors and small molecules to activate developmental signaling pathways and transcription factor networks. Through staged induction, pancreatic progenitor cells and endocrine progenitor cells eventually differentiate into mature endocrine cells (α, β, δ) cells (36–39). D’Amour et al. first attempted to establish a protocol for generating hormone-expressing cells that can synthesize and release multiple hormones (40). Rezania et al. reported a seven-step differentiation protocol, in which the resulting cells expressed key markers of mature β-cells, such as MAFA, PDX1, NKX6.1, and INS, and exhibited similar functionality to human islets (**Figure 2**) (41). Subsequently, Pagliuca et al. utilized human ESCs and employed a stepwise induction method with the addition of various factors in basal medium to successfully cultivate insulin-secreting β-cells (SC-β-cells), which functioned as fully functional pancreatic β-cells. Upon transplantation into mice, SC-β-cells showed detectable insulin secretion within two weeks, with secretion levels changing in response to blood glucose levels (42, 43). Directed differentiation protocols have also been reported for iPSCs, enabling the generation of cells expressing insulin and other mature β-cell markers (44, 45).

Because ESCs/iPSCs have good proliferation and differentiation ability and can produce large numbers of cells, they are ideal candidates for differentiation into islet β-cells and have broad application prospects for treating T1D. Therefore, how to produce SC-β-cells *in vitro* in large quantities has become the focus of research. The key transcription factors for differentiating ESCs/iPSCs into SC-β-cells *in vitro* are PDX1 and NKX6.1, both of which are highly expressed in pancreatic progenitor cells and required for producing monohormone, glucose-reactive β-cells (46, 47). Several research teams have reported differentiating ESCs/iPSCs into PDX1 and NKX6.1 co-expressing pancreatic progenitor cells (48, 49) in monolayer culture, and with improved experimental conditions, up to 90% of PDX1+/NKX6.1+ co-positive pancreatic progenitor cells were produced. Simultaneously, differentiating pancreatic progenitor cells into pancreatic β-cells has also made substantial progress, and the efficiency of pancreatic progenitor cells producing β-cells *in vitro* increased to about 40% (21, 42), although these β-cells are still different from human β-cells in other functions despite being responsive to glucose. To further improve the function of SC-beta cells, multiple research teams provided insights, such as Juan et al., who found that co-culturing with factors regulating circadian rhythm could enhance SC-beta cell function (50), Leonardo by altering the signaling pathway of SC-β-cells differentiation process (51), while Aharon et al. modified the nutrients in the medium used for *in vitro* differentiation to further enhance generating functional SC-β-cells *in vitro* (52), and could also induce SC-β-cells by simulating the 3D culture system of human pancreatic development (53). In addition, Isaura (54) and Mariana (55) et al. recently updated and detailed the recent progress in using ESCs/iPSCs derived islet β-cells *in vitro*. Some of the above protocols, although not reaching the level of the original human islet β-cells, promoted the development of stem cells differentiating into islet β-cells *in vitro*.

With the continuous development of stem cell technology, pancreatic β-cells products derived from ESCs/iPSCs are gradually used in clinical trials. A clinical trial conducted in 2014 (NCT02239354) used a stem cell-derived pancreatic endoderm cell population (PEC-01) developed by ViaCyte, Inc., which matured into insulin-producing endocrine cells *in vivo* over several months in animal models (56–58)., and in the clinical trials they have developed an immune protective device (PEC - Encap, VC - 01) is used to encapsulate PEC - 01, the device is a kind of biological membranes in order to eliminate the need for immunosuppression. The results of the trial showed that PEC-01 cell population could differentiate into β-cells and other islet cells after implantation under the patient’s skin, but excessive fibrosis around the device resulted in the end of the trial due to immune rejection (29, 30). To address this problem, the new device was modified with an opening in the biofilm that allowed vascularization, enhanced nutrient exchange so that host cells could also penetrate the device, and immunosuppressive therapy was administered after transplantation, while a more mature and functional cell population (PEC-02) was used. The results of a subsequent clinical trial (NCT03163511), published in 2021, showed that transplanted cells matured from pancreatic progenitor cells to pancreatic endocrine cells six months after transplantation, producing glucose-reactive C-peptide (59, 60) in six of the 17 patients who underwent the trial. Although the circulating C-peptide levels observed in these studies are still low, all demonstrate the potential of ESCs/iPSCs to differentiate into renewable islet β-cells. Most importantly, both studies, although in early stage clinical studies, did not identify any serious safety issues related to the transplanted cells, including tumor formation. ViaCyte was later acquired by Vertex. Another direction of clinical trials is transplanting fully differentiated SC-β-cells, which have been successful in non-human primates (61, 62). The most promising clinical trial to date is Vertex’s Phase I/II trial in 2021 (NCT04786262), which uses cells made of fully differentiated islet cells derived from pluripotent stem cells (VX-880) injected into the liver via a traditional portal route. Immunosuppressive therapy was also used to protect the transplanted islet cells from immune rejection. Some early results from the trial were recently published, with significant circulating C-peptide levels detected three months after transplantation and patients’ blood sugar significantly controlled, And well tolerated treatment (63). VX-880 is a novel stem cell derived product for the treatment of T1D, and the trial is continuing in the United States and Canada to further evaluate the safety and efficacy of the product. As the technology develops, more clinical trials are expected.

In addition to using ESC/iPSC-based techniques to induce the differentiation of transplantable β-cells *in vitro*, Zeng et al. proposed an alternative solution. Using single-cell sequencing technology, they discovered a previously unreported cell population in the mouse pancreas: protein C receptor-positive (Procr+) pancreatic cell population. These Procr+ endocrine progenitor cells can be cultured and induced to differentiate into islet-like cells. In a transplantation model of diabetic mice, transplanted islet-like organs reversed the disease (36). This finding provides a new direction for the direct extraction of target cells from the pancreas and induction of their differentiation into islet-like organs.

### 3.2 Islet cells of porcine islet origin

In addition to using stem cell-derived islet beta cells to replace donor islet cells, another potential option is xenotransplantation using porcine islets. Compared to human islets, pig reproduction is easier and pig islets are more readily available. More importantly, pig insulin is highly similar to human insulin, differing by only one amino acid. Pig insulin has been used to treat diabetes for decades. Pigs have organs similar in size to humans, enabling production of a sufficient number of islets for xenotransplantation. They are the most promising donor source for xenotransplantation. Although more porcine islet cells are required to achieve adequate insulin secretion compared to human donor islets, porcine islets appear to outperform human islets in studies. Porcine islet cell xenotransplantation has achieved insulin function in non-human primates, suggesting feasibility in clinical settings. Shin et al. reported long-term survival of adult porcine islets transplanted into five rhesus monkeys for over 20 months. These early trials suggest pig islets have great potential to address donor islet shortages for T1D patients.

However, there are still some urgent problems to be solved in the use of porcine islet xenotransplantation, the first of which is graft rejection. For example, infusion of porcine islets into the portal vein leads to activation of complement and clotting pathways, resulting in platelet aggregation and thrombosis at the transplant site and hyperacute rejection (64). This is followed by human responses to porcine islet antigens (Galactose α1,3-galactose and N-Glycolylneuraminic acid), as well as zoonotic infections caused by endogenous retroviruses.

In the meantime, solutions are being tried. One strategy is encapsulating islet cells without immunosuppression to solve the immune rejection problem in porcine islet xenotransplantation. Various natural or synthetic biomaterials are used for encapsulation, such as polyethylene glycol diacrylate (PEG-DA) (65), agarose (66), and other biological materials like alginate (67). Coating islet cells with alginate films containing polyethylene glycol acrylate has allowed survival up to 6 months without immunosuppression (68, 69). However, encapsulation risks hypoxia and nutrient deficiency in islet cells, delayed glucose and insulin diffusion affecting glucose regulation (70). One possible immunosuppression approach is co-stimulatory blocking. Studies in non-human primates showed anti-CD154 monoclonal antibodies combined with stimulus-blocking and standard immunization regimens injected through the portal vein prolonged transplanted porcine mice survival. However, no clinically available anti-CD154 monoclonal antibodies exist due to high thrombosis risk (71). We summarized relevant studies using immunosuppressive therapy to prolong porcine islet survival post-transplantation in **Table 2**. Technological developments like gene editing technologies like CRISPR/Cas9 potentially eliminate endogenous viruses in pigs, improving porcine islet xenotransplantation safety to humans (31). Gene editing overexpresses or knocks out multiple genes finding the best transgenic pigs for islet transplantation, avoiding xenotransplantation rejection (72). Recent studies showed targeted controlled mutational events successfully generated in pig cells through nuclease-directed homologous recombination (32).

In general, various differentiation protocols are available to induce the transformation of ESCs/iPSCs into insulin-producing cells. Clinical trial results have shown its safety and tolerance, making it a hot topic in current research with broad application prospects. However, the approach of directly selecting cells from the pancreatic tissue to induce pancreatic-like organs should not be abandoned. Finally, although extensive data on pig islets are still required from nonhuman primates for safety validation before clinical trials, they have gained popularity among many researchers. These different sources of β-cell replacement provide abundant choices for future clinical applications, allowing personalized treatment plans based on individual patient conditions. We summarized the advantages and disadvantages of using ESCs/iPSCs derived islet β-cells and porcine islet instead of β-cells as shown in **Table 3**.

**Table 3:** Comparison between SC-β-cells and porcine islets for the imminent cure of T1D.

Type	Advantages	Disadvantages
SC-β-cells	• The ability to proliferate and differentiate indefinitely • Easy to genetically engineer • Potential for standardized industrial production • Encapsulation reduces immune rejection	• Difficulty *in vitro* differentiation • Lower functional performance of stem cell-derived islet cells compared to primary human islets • Use of different pluripotent stem cell lines and protocols
Porcine islets	• Easy access to islet donors • Functionally similar to human pancreatic islets • Successful trials in non-human primates • Encapsulation to render long-term function	• Immune rejection due to xenotransplantation • Zoonotic infections caused by endogenous retroviruses • Porcine islet antigen

## 4 Ongoing challenges of islet transplantation immunosuppression

**Figure 3:**
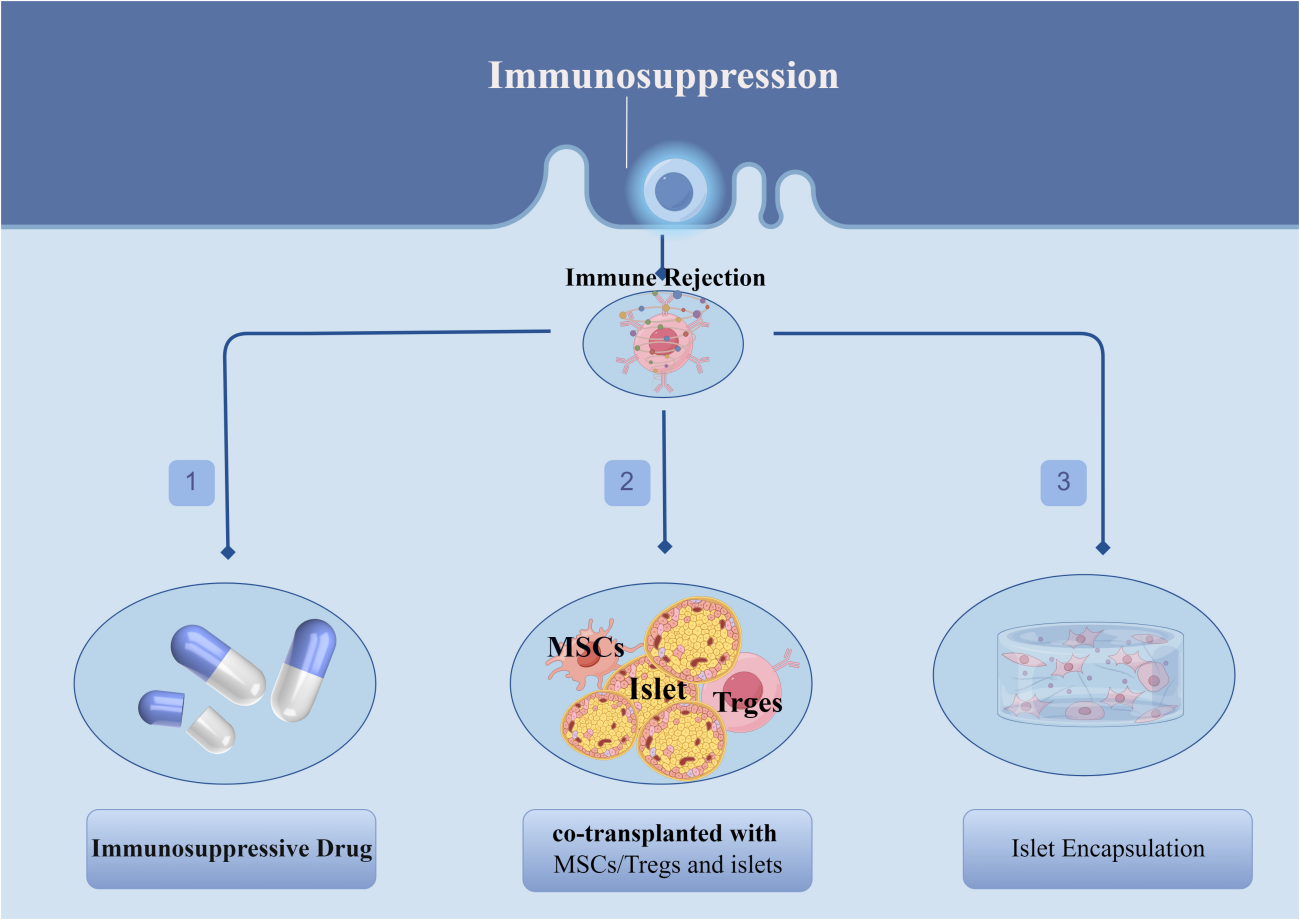
Different protocols for dealing with immune rejection after islet transplantation.

One of the greatest challenges that currently exists with islet transplantation is the post-transplant-induced recipient immune rejection, which may be responsible for the progressive decline in islet function in the years following islet transplantation as well as the inability of some patients to completely wean themselves from exogenous insulin therapy. These immune reactions include, but are not limited to: blood-mediated immediate inflammatory response (IBMIR) (73), recurrent autoimmune reactions (74, 75), and allogeneic rejection (76–78). Therefore, there is a clinical need to use high-quality islets from multiple donors or multiple inputs to counteract the substantial cell loss that occurs after transplantation (79). Currently, in order to overcome immune rejection after islet transplantation, in addition to the application of immunosuppressive drug, other new options have been explored, the most promising of which include the combined transplantation of mesenchymal stem cells (MSCs)/regulatory T cells (Tregs) and islet cells as well as the application of islet encapsulation techniques (**Figure 3**).

### 4.1 MSCs/Tregs were co-transplanted with islet cells

Mesenchymal stem cells (MSC),also known as stromal cells or mesenchymal progenitor cells, are a kind of non-hematopoietic stem cells derived from mesoderm, with multi-directional differentiation potential and strong self-renewal ability (80). MSC is relatively easy to obtain, can be obtained from human and rodent peripheral blood, placental tissue, umbilical cord blood, bone marrow cavity tissue and adipose tissue and other tissues and organs, and can be expanded and induced to differentiate *in vitro*, so it has been widely concerned and applied in the field of tissue engineering and regeneration. MSCs can improve the efficacy of IT in animal models, especially in regulating immune responses and protecting islet transplants (81–83). MSCs can improve insulin resistance in peripheral tissues through potential immunomodulatory and anti-inflammatory effects and promote pancreatic β-cell regeneration and protection (84, 85). Multiple studies have shown that, when co-cultured or co-transplanted with islet cells, MSCs can protect islet cells from apoptosis due to hypoxia and inflammatory cytokines through their secretory function, thus improving the survival of islet grafts *in vivo* and promoting the early recovery of the islet function (86, 87). In 2021, Kenyon et al. reported that islet cells and MSCs could be co-transplanted in non-human primate IT experiments. The results showed that the rejection-free survival and overall survival of treated islet grafts were significantly extended (88). Wang et al. used engineered MSCs as helper cells for islet co-transplantation and obtained similar results in diabetic mice. MSCs can induce local immune regulation and are potentially suitable for IT (89). Another study in patients with chronic pancreatitis showed that co-transplantation of autologous MSCs and islets is a safe and potential strategy for improving the islet function after transplantation (90). Generally speaking, co-transplantation with islet cells, it was found that mesenchymal stem cells had the functions of nutrition, support and protection to islet β-cells, as well as anti-inflammatory and immune regulation.

Regulatory T cells constitute a subset of T cells characterized by the presence of typical biological markers such as CD4+CD25+FoxP3+. These cells wield potent immunomodulatory functions and are pivotal in regulating immune homeostasis, upholding self-tolerance, and preventing excessive activation of the immune system (91). Tregs are considered a promising alternative to pharmacological agents that promote the engraftment and survival of transplanted organs/tissues (92–94). Tregs mainly produce self-tolerance, tolerance to alloantigens, and transplantation tolerance by inhibiting the activation and function of reactive effector T-cells (94). Currently, Treg therapy can be applied in two situations in IT: to promote the survival of islets during the initial transplantation and to induce peripheral tolerance to eliminate immunosuppression. The addition of Tregs at the time of islet infusion has been explored as a method to reduce the initial islet graft loss and improve islet engraftment (95–97). It has been reported that, in clinical models, Treg expansion *in vitro* and subsequent reinjection into patients can induce long-term remission of T1DM (98, 99). Although there are few relevant reports, a large amount of preclinical evidence shows that Treg-based treatment has benefits (100–102). Zielinski et al. recently reported a two-year study using a combined infusion of Tregs and rituximab to treat pediatric patients with T1DM. The study results show that combination therapy can delay disease progression compared with Treg or rituximab alone, and patients who received combination therapy were able to maintain higher insulin sensitivity and fasting C-peptide levels than patients in the single-treatment and control groups. Furthermore, patients who received Tregs alone had higher C-peptide levels than those in the untreated control group. Another ongoing clinical trial (NCT03182426) is observing the benefits of T cell depletion and dual anti-inflammatory treatment. If successful, it will provide new benefits to islet transplant patients.

With the development of IT, most traditional immunosuppressive drugs require continuous medication and cannot completely solve the problem of immune rejection in islet transplants. Cell therapy co-transplanted with MSCs/Tregs and islets has shown great advantages, although it is still in the experimental stage, and its application scenarios are broad.

### 4.2 Islet encapsulation

Islet encapsulation represents a promising approach to tackle host immune rejection, employing biomaterials to envelop islets in a protective barrier. This allows oxygen and nutrients to permeate islet cells while enabling secreted insulin to disseminate into the bloodstream. Concurrently, it shields islet cells from assault by the host immune system (103–105). This technology has developed rapidly over the past century and can be categorized into micro and macro-encapsulation based on different processes.

Micro-encapsulation technology encapsulates islets in a thin layer of biomaterials, facilitating exchange of nutrients, oxygen, and metabolites. Transplantation of these micro-encapsulated islets is also simplified. Alginate stands out as a particularly promising biomaterial due to its superior biocompatibility and ease of manufacture. Studies confirm alginate reduces post-transplantation immune rejection and enhances survival of encapsulated islet cells (105). For instance, incorporating chemokine CXCL12 into alginate micro-encapsulation protects islets and boosts islet cell function even without immunosuppressants (106). This alginate-based micro-encapsulation method has also been applied to encapsulate SC-β-cells, exhibiting no excessive fibrosis post-transplantation sans immunosuppressive therapy (107). It has emerged as a key biomaterial for β-cell encapsulation studies. Recently, research teams have modified extracellular matrix (ECM) components into alginate, simulating the pancreatic microenvironment to safeguard coated islet cells from immune cell and inflammatory factor impacts while promoting insulin secretion by islet β-cells (108, 109). Nevertheless, several challenges persist in leveraging micro-encapsulation, especially post-implantation, presenting potential issues.

Another macro-encapsulation technique can prevent direct graft-host immune cell contact and spread, and enable easy removal of any post-transplantation safety issues, and evaluating graft efficacy at any time, unavailable with micro-encapsulation (110). Macro-encapsulation has combated host immune rejection but is limited by inadequate oxygen and nutrient exchange before blood vessel formation around the device (30). Adding vascular endothelial growth factor (VEGF) and pre-vascularization improved this (111, 112). Recently, Wang et al. developed a new device with immunoprotective hydrogel and thermoplastic silica gel-polycarbonate-polyurethane maintaining islet function for up to 200 days (113) in allogeneic rodent islet transplant models. Another macro-encapsulation type encapsulated SC-β cells with amphoteric modified alginate gel, reversing hyperglycemia for 238 days (114) post-implantation in severe combined immunodeficiency (SCID) mice. Many research teams are studying islet packaging, and we summarize recent progress in **Table 4**.

**Table 4:** Different strategies and biomaterials for islet encapsulation.

Encapsulation material	Result	References
Carboxymethyl cellulose coated chitosan (CS@CMC) microgels	Long-term glucose regulation for 180 days was achieved in post-transplant diabetic mice	(115)
Methacrylated gelatin (GelMA), methacrylated heparin (HepMA) and VEGF	Reversed blood sugar levels in diabetic mice from high to normal blood sugar for at least 90 days	(116)
Zwitterionically modified alginate hydrogel	Hyperglycemia was reversed in SCID mice for 238 days	(114)
Immunoprotective hydrogel core and thermoplastic silicone-polycarbonate-urethane	In an allogeneic rodent islet transplantation model, use of the device was shown to maintain islet function for up to 200 days	(113)
Polytetrafluorethylene (PTFE)-membrane	Exhibit a rapid, vaso-independent and glucose-stimulated insulin response, early improvement of hyperglycemia and reduced fibrosis	(117)
Silicon nanopore membranes	Islets encapsulated with this device exhibit a highly active and biphasic insulin response to dynamic glucose stimulation	(118)
PTFE	After implantation, the patient experienced increased fasting C-peptide levels, increased glucose-reactive C-peptide levels, and mixed diet-stimulated C-peptide secretion.	(59)
Polyethylene glycol diacrylate (PEGDA)	The absence of immunosuppression reverses the signs of diabetes and leads to insulin-independent status or significantly reduced insulin requirements	(119)
Polyethylenglycol (PEG)	Reverse diabetes and maintain normal blood sugar for more than 80 days	(120)

### 4.3 Optimal transplant site

Currently, most clinical IT methods involve injecting islet cells through the hepatic portal vein under ultrasound guidance. This is a conventional, mature method (11, 121). However, portal vein IT can cause postoperative bleeding, vascular emboli formation, portal hypertension, and periportal fatty degeneration. In particular, the blood-mediated acute inflammatory response (IBMIR) caused by portal vein transplantation can result in massive graft loss in the very early stages of transplantation (122), suggesting that the liver is not the most suitable site for IT. Researchers are exploring different organs and sites (**Figure 2B**) to determine the best location for islet cell transplantation (**Table 5**).

**Table 5:** Selection of transplantation sites other than the liver.

Transplantation sites	Receptor	Bio-materials	Result	References
Omentum	Diabetic rats	Hydrogels	Transplanted pancreatic islets show high rates of peri-islet and intra-islet hemotransfusion and reverse diabetes	(123)
	T1D patient	Biocompatible Plasma-Thrombin Gel	Stable glycemic control over 9 months, but relapse after 1 year	(124)
	Lewis rats	Plasma-thrombin bioscaffold	Maintained normal blood glucose for 100 days post-transplant	(125)
Intramuscular	7 years old patient		Quality of life improves, but exogenous insulin is still needed	(126)
	Lewis rats		Significantly lower blood sugar levels after islet transplantation	(127)
Subcutaneous space	Diabetic mice	Biodegradable temporizing matrix	Porcine islet cells survive more than 100 days after transplantation and secrete C-peptide	(128)
	Diabetic mice	Methacrylic acid-polyethylene glycol	Reversal of diabetes by injection of 600 rodent islet equivalents for 70 days	(129)
Anterior Chamber of the Eye (ACE)	Baboon		Decreased exogenous insulin requirement, no serious adverse effects seen	(130)

The omentum represents a potentially valuable transplant site, offering avoidance of IBMIR compared to traditional portal vein inflow. This richly vascularized tissue secretes various growth factors (e.g. CXCR4, VEGF, and SDF-1) that promote islet vascularization and survival (131, 132). In addition, omentum possesses immunomodulatory capabilities and can monitor the graft for prompt removal if adverse reactions occur. Omental transplantation using biological scaffolds has been used for clinical applications. A US trial (NCT02213003) transplanted pancreatic islets into the omentum of T1DM patients (133). Insulin independence was achieved by day 17 post-transplant but declined approximately one year later. Another ongoing trial (NCT02821026) has shown limited success. However, in 2023, Deng et al. reported a method of omental allogeneic IT in nonhuman primates using locally applied recombinant thrombin (Recothrom) and the recipient’s autologous plasma to design a degradable matrix for islet fixation. Normal blood sugar and insulin independence were achieved at one week post-transplant, with stable expression thereafter. This study provides strategies for the clinical translation of omental transplantation.

The subcutaneous space is another ideal transplant site. It is a relatively avascular region that is easily accessible to biomaterials or macroscopically encapsulated islets. In 2020, Yu et al. reported successful subcutaneous IT in various immune-competent and immune-naïve animal models using a device-free islet survival matrix to achieve long-term normoglycemia. This method has been used for mice, pigs, and humans. Islet cell transplant models have the advantages of simplicity, safety, and reproducibility (134). With the clinical application of ESCs/iPSC-derived islet-like cells and islet encapsulation technology, the subcutaneous cavity can be easily monitored and removed, making it a promising transplant method. However, the skin lacks relative blood vessels and cannot obtain early-stage nutrients and oxygen, which limits its clinical application. To address this, Darling et al. tested a biodegradable temporary matrix based on a polyurethane scaffold that forms good blood vessels within the skin. In a porcine islet transplant model, grafts maintained normal function and survived for over three months (128). In addition, the immune response hinders subcutaneous transplantation. Therefore, the development of advanced biomaterials with angiogenesis and immune modulation capabilities may be the next step for the long-term islet survival and function in the skin.

In addition to the two aforementioned research hotspots of transplant sites, studies on transplanting islets into the intrapleural (135), skeletal muscle (136), anterior chamber of the eye (ACE) (137), and other sites have been reported (138–140). However, research on these aspects is still in its infancy, and there is a large gap in clinical applications. Due to the application of bioengineering materials and macro-encapsulated islet grafts, the greater omentum and subcutaneous space seem to be ideal sites for IT in the future.

### 4.4 Immunosuppression

Although several of the above options are effective in mitigating the immune rejection caused by islet transplantation and are the way forward, immunosuppressive therapy is still required at this time to ensure islet survival and function. The goal of immunosuppression is to provide effective and sustained immune protection in the smallest effective amount without suffering from the side effects associated with immunosuppression. Since inflammation leads to significant islet loss, anti-inflammatory drugs reduce damage from pro-inflammatory factors and may improve islet cell function in the early post-transplant period (141). Therefore, in order to attenuate the IBMIR response that occurs after islet transplantation and thereby reduce islet loss, several anti-inflammatory therapies have been used in the perioperative period of islet transplantation, including TNF-α inhibitors (etanercept), IL-1 receptor antagonists (anabolic acid), and α1-antitrypsin. Enalcipro, which targets TNF-α, is a potent antitumor agent that is widely used in T1D patients with allogeneic transplantation (17), and its use in mouse animal models results in a reduction of inflammatory markers and has been shown to have a sustained effect on autoimmunity (142). And in another study in an immunodeficient mouse islet transplant model, it was found that the percentage of mice achieving normal blood glucose levels after transplantation with the combination of etanercept and anabolic acid was 87.5%, compared to 45.45% with etanercept alone, and 53.9% with anabolic acid alone, suggesting that the combined use of etanercept and anabolic acid significantly improves the function of islet grafts (143). However, a recent study showed that although the use of etanercept demonstrated better islet function in the pre-transplant period, this advantage was not found to be sustained at the subsequent 1- or 2-year follow-up, and therefore, different doses or prolonged use of etanercept need to be explored to benefit patients (144). Another promising anti-inflammatory is α1-antitrypsin, which is a serine protease inhibitor, has been shown in several preclinical studies in animal islet transplantation models to attenuate the IBMIR response and prevent islet cell apoptosis while inhibiting cytokine-induced islet inflammatory responses (145, 146).

## 5 Conclusion and outlook

In terms of long-term results of islet transplantation, this study has greatly advanced research in the treatment of diabetes, and optimized protocols for long-term efficacy of islet transplantation have demonstrated the superiority of this approach, eliminating the dependence on exogenous insulin in a significant proportion of patients, thus avoiding diabetes-related complications. However islet transplantation still faces challenges such as shortage of islet sources and immunosuppression. To address the shortage of islet donors, we highlight stem cell-derived pancreatic β-cells and porcine islets as future solutions. Where stem cells are differentiated *in vitro* to generate pancreatic β-cells are being investigated for more efficient differentiation protocols, cell culture expansion methods and islet encapsulation techniques to optimize production to provide protection against the patient’s autoimmune response. Porcine islet xenotransplantation is becoming a reality and if successful will provide a constant supply of high quality islet donors, however, xenoantigens and strong immunosuppressive responses are currently the main challenges and gene editing using CRISPR-Cas9 is expected to bring a brighter future for porcine islet xenotransplantation. In addition to overcome the immunosuppression, islet encapsulation technology is currently being developed, and various encapsulation materials: natural or synthetic biomaterials are showing clear advantages in several preclinical and clinical trials, and although the ideal biocompatible material is still a matter of debate, it is undeniable that islet encapsulation technology provides a barrier to protect transplanted islets, and in the future it will be mainly useful in preventing hyperfibrosis, promoting local vascularization, and preventing the emergence of chronic immunosuppressive rejection. MSCs/Tregs and islet cell co-transplantation shows a broader prospect, which can minimize the use of immunosuppressant and reduce the side effects of immunosuppressant once it is successfully applied. Since islet grafts do not survive long term after portal vein infusion, which suggests that this site is not the optimal site for islet transplantation, subcutaneous lumen and greater omentum based encapsulation device is a more attractive strategy in comparison. Not only does it provide a physical barrier that reduces the destruction of the transplanted islets by the body’s immune cells, thereby improving islet survival and function. At the same time, this strategy can be adapted as needed, such as removing the device in the event of an adverse reaction, and this flexibility can also be applied to individualize treatment as the patient’s specific needs evolve.

The recent advent of single-cell sequencing technology (scRNA-seq) has ushered in a new era of molecular dissection, which is capable of revealing differential gene expression at the level of individual cells (147). In the field of islet transplantation, scRNA-seq may help to reveal the characteristics of different cell types in allogeneic islet transplants and be able to pinpoint cellular stress responses and pathophysiological changes in different grafts, which may further prolong islet graft survival and functional improvement, ultimately leading to insulin independence (148). In conclusion, with the innovative research carried out on islet source acquisition, immunosuppression protocols, and graft site reselection for islet transplantation, this technology will certainly be driven to greater maturity.

## Author contributions

QW: Writing – original draft. Y-xH: Writing – original draft. LL: Writing – original draft. X-hZ: Writing – original draft. YS: Writing – original draft. XM: Writing – review & editing. S-wL: Writing – original draft, Writing – review & editing.

## References

[1] Atkinson MA Eisenbarth GS Michels AW. Type 1 diabetes. Lancet. (2014) 383:69–82. doi: 10.1016/S0140-6736(13)60591-7 23890997 PMC4380133

[2] Tripathi BK Srivastava AK. Diabetes mellitus: complications and therapeutics. Med Sci Monit. (2006) 12:RA130–47. 16810145

[3] Bergenstal RM Tamborlane WV Ahmann A Buse JB Dailey G Davis SN. Effectiveness of sensor-augmented insulin-pump therapy in type 1 diabetes. N Engl J Med. (2010) 363:311–20. doi: 10.1056/NEJMoa1002853 20587585

[4] Karges B Binder E Rosenbauer J. Complications with insulin pump therapy vs insulin injection therapy-reply. JAMA. (2018) 319:503–4. doi: 10.1001/jama.2017.20357 29411030

[5] Shapiro AM Pokrywczynska M Ricordi C. Clinical pancreatic islet transplantation. Nat Rev Endocrinol. (2017) 13:268–77. doi: 10.1038/nrendo.2016.178 27834384

[6] Marfil-Garza BA Shapiro AMJ Kin T. Clinical islet transplantation: Current progress and new frontiers. J Hepatobiliary Pancreat Sci. (2021) 28:243–54. doi: 10.1002/jhbp.891 33417749

[7] Israni AK Zaun D Rosendale JD Snyder JJ Kasiske BL. OPTN/SRTR 2012 Annual Data Report: deceased organ donation. Am J Transplant. (2014) 14 Suppl 1:167–83. doi: 10.1111/ajt.12585 24373172

[8] Israni AK Skeans MA Gustafson SK Schnitzler MA Wainright JL Carrico RJ. OPTN/SRTR 2012 annual data report: pancreas. Am J Transplant. (2014) 14 Suppl 1:45–68. doi: 10.1111/ajt.12580 24373167

[9] Shapiro AM. Islet transplantation in type 1 diabetes: ongoing challenges, refined procedures, and long-term outcome. Rev Diabetes Stud. (2012) 9:385–406. doi: 10.1900/RDS.2012.9.385 PMC3740705 23804275

[10] Sutherland DE Gores PF Farney AC Wahoff DC Matas AJ Dunn DL. Evolution of kidney, pancreas, and islet transplantation for patients with diabetes at the University of Minnesota. Am J Surg. (1993) 166:456–91. doi: 10.1016/s0002-9610(05)81142-0 8238742

[11] Shapiro AM Lakey JR Ryan EA Korbutt GS Toth E Warnock GL. Islet transplantation in seven patients with type 1 diabetes mellitus using a glucocorticoid-free immunosuppressive regimen. N Engl J Med. (2000) 343:230–8. doi: 10.1056/NEJM200007273430401 10911004

[12] Shapiro AM Ricordi C Hering BJ Auchincloss H Lindblad R Robertson RP. International trial of the Edmonton protocol for islet transplantation. N Engl J Med. (2006) 355:1318–30. doi: 10.1056/NEJMoa061267 17005949

[13] Markmann JF Deng S Huang X Desai NM Velidedeoglu EH Lui C. Insulin independence following isolated islet transplantation and single islet infusions. Ann Surg. (2003) 237:741–50. doi: 10.1097/01.SLA.0000072110.93780.52 PMC1514687 12796569

[14] Posselt AM Szot GL Frassetto LA Masharani U Tavakol M Amin R. Islet transplantation in type 1 diabetic patients using calcineurin inhibitor-free immunosuppressive protocols based on T-cell adhesion or costimulation blockade. Transplantation. (2010) 90:1595–601. doi: 10.1097/TP.0b013e3181fe1377 PMC4296579 20978464

[15] Posselt AM Bellin MD Tavakol M Szot GL Frassetto LA Masharani U. Islet transplantation in type 1 diabetics using an immunosuppressive protocol based on the anti-LFA-1 antibody efalizumab. Am J Transplant. (2010) 10:1870–80. doi: 10.1111/j.1600-6143.2010.03073.x PMC2911648 20659093

[16] Hering BJ Kandaswamy R Harmon JV Ansite JD Clemmings SM Sakai T. Transplantation of cultured islets from two-layer preserved pancreases in type 1 diabetes with anti-CD3 antibody. Am J Transplant. (2004) 4:390–401. doi: 10.1046/j.1600-6143.2003.00351.x 14961992

[17] Hering BJ Kandaswamy R Ansite JD Eckman PM Nakano M Sawada T. Single-donor, marginal-dose islet transplantation in patients with type 1 diabetes. JAMA. (2005) 293:830–5. doi: 10.1001/jama.293.7.830 15713772

[18] Gangemi A Salehi P Hatipoglu B Martellotto J Barbaro B Kuechle JB. Islet transplantation for brittle type 1 diabetes: the UIC protocol. Am J Transplant. (2008) 8:1250–61. doi: 10.1111/j.1600-6143.2008.02234.x 18444920

[19] Matsumoto S Takita M Chaussabel D Noguchi Shimoda H Sugimoto M K. Improving efficacy of clinical islet transplantation with iodixanol-based islet purification, thymoglobulin induction, and blockage of IL-1β and TNF-α. Cell Transplant. (2011) 20:1641–7. doi: 10.3727/096368910X564058 21396171

[20] Maffi P Berney T Nano R Niclauss N Bosco D Melzi R. Calcineurin inhibitor-free immunosuppressive regimen in type 1 diabetes patients receiving islet transplantation: single-group phase 1/2 trial. Transplantation. (2014) 98:1301–9. doi: 10.1097/TP.0000000000000396 25286053

[21] Hering BJ Ballou CM Bellin MD Payne EH Kandeel F Witkowski P. Factors associated with favourable 5 year outcomes in islet transplant alone recipients with type 1 diabetes complicated by severe hypoglycaemia in the Collaborative Islet Transplant Registry. Diabetologia. (2023) 66:163–73. doi: 10.1007/s00125-022-05804-4 PMC10355148 36201044

[22] Nijhoff MF Engelse MA Dubbeld J Braat AE Ringers J Roelen DL. Glycemic stability through islet-after-kidney transplantation using an alemtuzumab-based induction regimen and long-term triple-maintenance immunosuppression. Am J Transplant. (2016) 16:246–53. doi: 10.1111/ajt.13425 26288226

[23] Cardona K Korbutt GS Milas Z Lyon J Cano J Jiang W. Long-term survival of neonatal porcine islets in nonhuman primates by targeting costimulation pathways. Nat Med. (2006) 12:304–6. doi: 10.1038/nm1375 16501570

[24] Hering BJ Wijkstrom M Graham ML Hårdstedt M Aasheim TC Jie T. Prolonged diabetes reversal after intraportal xenotransplantation of wild-type porcine islets in immunosuppressed nonhuman primates. Nat Med. (2006) 12:301–3. doi: 10.1038/nm1369 16491083

[25] Thompson P Cardona K Russell M Badell IR Shaffer V Korbutt G. CD40-specific costimulation blockade enhances neonatal porcine islet survival in nonhuman primates. Am J Transplant. (2011) 11:947–57. doi: 10.1111/j.1600-6143.2011.03509.x PMC4845096 21521467

[26] Thompson P Badell IR Lowe M Turner A Cano J Avila J. Alternative immunomodulatory strategies for xenotransplantation: CD40/154 pathway-sparing regimens promote xenograft survival. Am J Transplant. (2012) 12:1765–75. doi: 10.1111/j.1600-6143.2012.04031.x PMC3387302 22458586

[27] Shin JS Kim JM Kim JS Min BH Kim YH Kim HJ. Long-term control of diabetes in immunosuppressed nonhuman primates (NHP) by the transplantation of adult porcine islets. Am J Transplant. (2015) 15:2837–50. doi: 10.1111/ajt.13345 26096041

[28] Shin JS Kim JM Min BH Yoon IH Kim HJ Kim JS. Pre-clinical results in pig-to-non-human primate islet xenotransplantation using anti-CD40 antibody (2C10R4)-based immunosuppression. Xenotransplantation. (2018) 25(1):10.1111/xen.12356. doi: 10.1111/xen.12356 PMC5809197 29057561

[29] Agulnick AD Ambruzs DM Moorman MA Bhoumik A Cesario RM Payne JK. Insulin-producing endocrine cells differentiated *in vitro* from human embryonic stem cells function in macroencapsulation devices *in vivo* . Stem Cells Transl Med. (2015) 4:1214–22. doi: 10.5966/sctm.2015-0079 PMC4572906 26304037

[30] Dolgin E. Diabetes: encapsulating the problem. Nature. (2016) 540:S60–2. doi: 10.1038/540S60a 27926697

[31] Yang L Güell M Niu D George H Lesha E Grishin D. Genome-wide inactivation of porcine endogenous retroviruses (PERVs). Science. (2015) 350:1101–4. doi: 10.1126/science.aad1191 26456528

[32] Butler JR Santos RMN Martens GR Ladowski JM Wang ZY Li P. Efficient generation of targeted and controlled mutational events in porcine cells using nuclease-directed homologous recombination. J Surg Res. (2017) 212:238–45. doi: 10.1016/j.jss.2017.01.025 28550913

[33] Hering BJ Clarke WR Bridges ND Eggerman TL Alejandro R Bellin MD. Phase 3 trial of transplantation of human islets in type 1 diabetes complicated by severe hypoglycemia. Diabetes Care. (2016) 39:1230–40. doi: 10.2337/dc15-1988 PMC5317236 27208344

[34] Marfil-Garza BA Imes S Verhoeff K Hefler J Lam A Dajani K. Pancreatic islet transplantation in type 1 diabetes: 20-year experience from a single-centre cohort in Canada. Lancet Diabetes Endocrinol. (2022) 10:519–32. doi: 10.1016/S2213-8587(22)00114-0 35588757

[35] Chetboun M Drumez E Ballou C Maanaoui M Payne E Barton F. Association between primary graft function and 5-year outcomes of islet allogeneic transplantation in type 1 diabetes: a retrospective, multicentre, observational cohort study in 1210 patients from the Collaborative Islet Transplant Registry. Lancet Diabetes Endocrinol. (2023) 11:391–401. doi: 10.1016/S2213-8587(23)00082-7 37105208 PMC10388704

[36] Wang D Wang J Bai L Pan H Feng H Clevers H. Long-term expansion of pancreatic islet organoids from resident procr+ Progenitors. Cell. (2020) 180:1198–1211.e19. doi: 10.1016/j.cell.2020.02.048 32200801

[37] Li W Nakanishi M Zumsteg A Shear M Wright C Melton DA. *In vivo* reprogramming of pancreatic acinar cells to three islet endocrine subtypes. Elife. (2014) 3:e01846. doi: 10.7554/eLife.01846 24714494 PMC3977343

[38] Rodriguez-Diaz R Molano RD Weitz JR Abdulreda MH Berman DM Leibiger B. Paracrine interactions within the pancreatic islet determine the glycemic set point. Cell Metab. (2018) 27:549–558.e4. doi: 10.1016/j.cmet.2018.01.015 29514065 PMC5872154

[39] Hart NJ Powers AC. Use of human islets to understand islet biology and diabetes: progress, challenges and suggestions. Diabetologia. (2019) 62:212–22. doi: 10.1007/s00125-018-4772-2 PMC6325002 30547228

[40] D’Amour KA Bang AG Eliazer S Kelly OG Agulnick AD Smart NG. Production of pancreatic hormone-expressing endocrine cells from human embryonic stem cells. Nat Biotechnol. (2006) 24:1392–401. doi: 10.1038/nbt1259 17053790

[41] Rezania A Bruin JE Arora P Rubin A Batushansky I Asadi A. Reversal of diabetes with insulin-producing cells derived *in vitro* from human pluripotent stem cells. Nat Biotechnol. (2014) 32:1121–33. doi: 10.1038/nbt.3033 25211370

[42] Pagliuca FW Millman JR Gürtler M Segel M Van Dervort A Ryu JH. Generation of functional human pancreatic β-cells *in vitro* . Cell. (2014) 159:428–39. doi: 10.1016/j.cell.2014.09.040 PMC4617632 25303535

[43] Vegas AJ Veiseh O Gürtler M Millman JR Pagliuca FW Bader AR. Long-term glycemic control using polymer-encapsulated human stem cell-derived β-cells in immune-competent mice. Nat Med. (2016) 22:306–11. doi: 10.1038/nm.4030 PMC4825868 26808346

[44] Pagliuca FW Melton DA. How to make a functional β-cell. Development. (2013) 140:2472–83. doi: 10.1242/dev.093187 PMC3666377 23715541

[45] Rostovskaya M Bredenkamp N Smith A. Towards consistent generation of pancreatic lineage progenitors from human pluripotent stem cells. Philos Trans R Soc Lond B Biol Sci. (2015) 370:20140365. doi: 10.1098/rstb.2014.0365 26416676 PMC4633994

[46] Rezania A Bruin JE Xu J Narayan K Fox JK O'Neil JJ. Enrichment of human embryonic stem cell-derived NKX6.1-expressing pancreatic progenitor cells accelerates the maturation of insulin-secreting cells *in vivo* . Stem Cells. (2013) 31:2432–42. doi: 10.1002/stem.1489 23897760

[47] Jennings RE Berry AA Kirkwood-Wilson R Roberts NA Hearn T Salisbury RJ. Development of the human pancreas from foregut to endocrine commitment. Diabetes. (2013) 62:3514–22. doi: 10.2337/db12-1479 PMC3781486 23630303

[48] Nostro MC Sarangi F Yang C Holland A Elefanty AG Stanley EG. Efficient generation of NKX6–1+ pancreatic progenitors from multiple human pluripotent stem cell lines. Stem Cell Rep. (2015) 4:591–604. doi: 10.1016/j.stemcr.2015.02.017 PMC4400642 25843049

[49] Cogger KF Sinha A Sarangi F McGaugh EC Saunders D Dorrell C. Glycoprotein 2 is a specific cell surface marker of human pancreatic progenitors. Nat Commun. (2017) 8:331. doi: 10.1038/s41467-017-00561-0 28835709 PMC5569081

[50] Alvarez-Dominguez JR Donaghey J Rasouli N Kenty JHR Helman A Charlton J. Circadian entrainment triggers maturation of human *in vitro* islets. Cell Stem Cell. (2020) 26:108–122.e10. doi: 10.1016/j.stem.2019.11.011 31839570

[51] Velazco-Cruz L Song J Maxwell KG Goedegebuure MM Augsornworawat P Hogrebe NJ. Acquisition of dynamic function in human stem cell-derived β Cells. Stem Cell Rep. (2019) 12:351–65. doi: 10.1016/j.stemcr.2018.12.012 PMC6372986 30661993

[52] Helman A Cangelosi AL Davis JC Pham Q Rothman A Faust AL. A nutrient-sensing transition at birth triggers glucose-responsive insulin secretion. Cell Metab. (2020) 31:1004–1016.e5. doi: 10.1016/j.cmet.2020.04.004 32375022 PMC7480404

[53] Gonçalves CA Larsen M Jung S Stratmann J Nakamura A Leuschner M. A 3D system to model human pancreas development and its reference single-cell transcriptome atlas identify signaling pathways required for progenitor expansion. Nat Commun. (2021) 12:3144. doi: 10.1038/s41467-021-23295-6 34035279 PMC8149728

[54] Silva IBB Kimura CH Colantoni VP Sogayar MC. Stem cells differentiation into insulin-producing cells (IPCs): recent advances and current challenges. Stem Cell Res Ther. (2022) 13:309. doi: 10.1186/s13287-022-03206-2 35840987 PMC9284809

[55] Karimova MV Gvazava IG Vorotelyak EA. Overcoming the limitations of stem cell-derived beta cells. Biomolecules. (2022) 12:810. doi: 10.3390/biom12060810 35740935 PMC9221417

[56] Kelly OG Chan MY Martinson LA Kadoya K Ostertag TM Ross KG. Cell-surface markers for the isolation of pancreatic cell types derived from human embryonic stem cells. Nat Biotechnol. (2011) 29:750–6. doi: 10.1038/nbt.1931 21804561

[57] Rezania A Bruin JE Riedel MJ Mojibian M Asadi A Xu J. Maturation of human embryonic stem cell-derived pancreatic progenitors into functional islets capable of treating pre-existing diabetes in mice. Diabetes. (2012) 61:2016–29. doi: 10.2337/db11-1711 PMC3402300 22740171

[58] Kroon E Martinson LA Kadoya K Bang AG Kelly OG Eliazer S. Pancreatic endoderm derived from human embryonic stem cells generates glucose-responsive insulin-secreting cells *in vivo* . Nat Biotechnol. (2008) 26:443–52. doi: 10.1038/nbt1393 18288110

[59] Ramzy A Thompson DM Ward-Hartstonge KA Ivison S Cook L Garcia RV. Implanted pluripotent stem-cell-derived pancreatic endoderm cells secrete glucose-responsive C-peptide in patients with type 1 diabetes. Cell Stem Cell. (2021) 28:2047–2061.e5. doi: 10.1016/j.stem.2021.10.003 34861146

[60] Shapiro AMJ Thompson D Donner TW Bellin MD Hsueh W Pettus J. Insulin expression and C-peptide in type 1 diabetes subjects implanted with stem cell-derived pancreatic endoderm cells in an encapsulation device. Cell Rep Med. (2021) 2:100466. doi: 10.1016/j.xcrm.2021.100466 35028608 PMC8714853

[61] Du Y Liang Z Wang S Sun D Wang X Liew SY. Human pluripotent stem-cell-derived islets ameliorate diabetes in non-human primates. Nat Med. (2022) 28:272–82. doi: 10.1038/s41591-021-01645-7 35115708

[62] Liang Z Sun D Lu S Lei Z Wang S Luo Z. Implantation underneath the abdominal anterior rectus sheath enables effective and functional engraftment of stem-cell-derived islets. Nat Metab. (2023) 5:29–40. doi: 10.1038/s42255-022-00713-7 36624157

[63] Vertex. Vertex announces positive day 90 data for the frst patient in the phase 1/2 clinical trial dosed with VX-880, a Novel investigational stem cell-derived therapy for the treatment of type 1 diabetes . Available online at: https://news.vrtx.com/press-release/vertex-announces-positive-day-90-data-frstpatient-phase-12-clinical-trial-dosed-vx?_ga=2.53361578.345811804.1646342387–705593813.1646342387.

[64] Ekser B Cooper DK. Overcoming the barriers to xenotransplantation: prospects for the future. Expert Rev Clin Immunol. (2010) 6:219–30. doi: 10.1586/eci.09.81 PMC2857338 20402385

[65] Cruise GM Hegre OD Lamberti FV Hager SR Hill R Scharp DS. *In vitro* and *in vivo* performance of porcine islets encapsulated in interfacially photopolymerized poly(ethylene glycol) diacrylate membranes. Cell Transplant. (1999) 8:293–306. doi: 10.1177/096368979900800310 10442742

[66] Gazda LS Vinerean HV Laramore MA Hall RD Carraway JW Smith BH. No evidence of viral transmission following long-term implantation of agarose encapsulated porcine islets in diabetic dogs. J Diabetes Res. (2014) 2014:727483. doi: 10.1155/2014/727483 24995342 PMC4068064

[67] Pasqua M Pereira U de Lartigue C Nicolas J Vigneron P Dermigny Q. Preclinical characterization of alginate-poly-L-lysine encapsulated HepaRG for extracorporeal liver supply. Biotechnol Bioeng. (2021) 118:453–64. doi: 10.1002/bit.27583 32997339

[68] Matsumoto S Abalovich A Wechsler C Wynyard S Elliott RB. Clinical benefit of islet xenotransplantation for the treatment of type 1 diabetes. EBioMedicine. (2016) 12:255–62. doi: 10.1016/j.ebiom.2016.08.034 PMC5078586 27592597

[69] Gianello P. Macroencapsulated pig islets correct induced diabetes in primates up to 6 months. Adv Exp Med Biol. (2015) 865:157–70. doi: 10.1007/978-3-319-18603-0_10 26306449

[70] Korsgren O. Islet encapsulation: physiological possibilities and limitations. Diabetes. (2017) 66:1748–54. doi: 10.2337/db17-0065 28637827

[71] Bottino R Knoll MF Graeme-Wilson J Klein EC Ayares D Trucco M. Safe use of anti-CD154 monoclonal antibody in pig islet xenotransplantation in monkeys. Xenotransplantation. (2017) 24(1):10.1111/xen.12283. doi: 10.1111/xen.12283 PMC5332295 28058735

[72] Cooper DKC Mou L Bottino R. A brief review of the current status of pig islet xenotransplantation. Front Immunol. (2024) 15:1366530. doi: 10.3389/fimmu.2024.1366530 38464515 PMC10920266

[73] Cheng Y Wang B Li H Zhao N Liu Y. Mechanism for the instant blood-mediated inflammatory reaction in rat islet transplantation. Transplant Proc. (2017) 49:1440–3. doi: 10.1016/j.transproceed.2017.03.090 28736020

[74] Pearson T Markees TG Serreze DV Pierce MA Wicker LS Peterson LB. Islet cell autoimmunity and transplantation tolerance: two distinct mechanisms? Ann N Y Acad Sci. (2003) 1005:148–56. doi: 10.1196/annals.1288.016 14679049

[75] Rossini AA Mordes JP Greiner DL Stoff JS. Islet cell transplantation tolerance. Transplantation. (2001) 72:S43–6. 11888156

[76] Eich T Eriksson O Lundgren T Nordic Network for Clinical Islet Transplantation. Visualization of early engraftment in clinical islet transplantation by positron-emission tomography. N Engl J Med. (2007) 356:2754–5. doi: 10.1056/NEJMc070201 17596618

[77] Eich T Eriksson O Sundin A Estrada S Brandhorst D Brandhorst H. Positron emission tomography: a real-time tool to quantify early islet engraftment in a preclinical large animal model. Transplantation. (2007) 84:893–8. doi: 10.1097/01.tp.0000284730.86567.9f 17984843

[78] Citro A Cantarelli E Piemonti L. Anti-inflammatory strategies to enhance islet engraftment and survival. Curr Diabetes Rep. (2013) 13:733–44. doi: 10.1007/s11892-013-0401-0 23912763

[79] McCall M Shapiro AM. Update on islet transplantation. Cold Spring Harb Perspect Med. (2012) 2:a007823. doi: 10.1101/cshperspect.a007823 22762022 PMC3385934

[80] Brown C McKee C Bakshi S Walker K Hakman E Halassy S. Mesenchymal stem cells: Cell therapy and regeneration potential. J Tissue Eng Regener Med. (2019) 13:1738–55. doi: 10.1002/term.2914 31216380

[81] Borg DJ Weigelt M Wilhelm C Gerlach M Bickle M Speier S. Mesenchymal stromal cells improve transplanted islet survival and islet function in a syngeneic mouse model. Diabetologia. (2014) 57:522–31. doi: 10.1007/s00125-013-3109-4 24253203

[82] Newton WC Kim JW Luo JZQ Luo L. Stem cell-derived exosomes: a novel vector for tissue repair and diabetic therapy. J Mol Endocrinol. (2017) 59:R155–65. doi: 10.1530/JME-17-0080 28835418

[83] Mou L Wang TB Wang X Pu Z. Advancing diabetes treatment: the role of mesenchymal stem cells in islet transplantation. Front Immunol. (2024) 15:1389134. doi: 10.3389/fimmu.2024.1389134 38605972 PMC11007079

[84] Shen J Cheng Y Han Q Mu Y Han W. Generating insulin-producing cells for diabetic therapy: existing strategies and new development. Ageing Res Rev. (2013) 12:469–78. doi: 10.1016/j.arr.2013.01.001 23318683

[85] Ortiz LA Gambelli F McBride C Gaupp D Baddoo M Kaminski N. Mesenchymal stem cell engraftment in lung is enhanced in response to bleomycin exposure and ameliorates its fibrotic effects. Proc Natl Acad Sci U S A. (2003) 100:8407–11. doi: 10.1073/pnas.1432929100 PMC166242 12815096

[86] Hubber EL Rackham CL Jones PM. Protecting islet functional viability using mesenchymal stromal cells. Stem Cells Transl Med. (2021) 10:674–80. doi: 10.1002/sctm.20-0466 PMC8046085 33544449

[87] Schive SW Mirlashari MR Hasvold G Wang M Josefsen D Gullestad HP. Human adipose-derived mesenchymal stem cells respond to short-term hypoxia by secreting factors beneficial for human islets *in vitro* and potentiate antidiabetic effect *in vivo* . Cell Med. (2017) 9:103–16. doi: 10.3727/215517917X693401 PMC5509020 28713640

[88] Kenyon NS Willman MA Han D Leeman RS Rabassa A Diaz WL. Extended survival versus accelerated rejection of nonhuman primate islet allografts: Effect of mesenchymal stem cell source and timing. Am J Transplant. (2021) 21:3524–37. doi: 10.1111/ajt.16693 PMC9034438 34008325

[89] Wang X Wang K Yu M Velluto D Hong X Wang B. Engineered immunomodulatory accessory cells improve experimental allogeneic islet transplantation without immunosuppression. Sci Adv. (2022) 8:eabn0071. doi: 10.1126/sciadv.abn0071 35867788 PMC9307254

[90] Wang H Strange C Nietert PJ Wang J Turnbull TL Cloud C. Autologous mesenchymal stem cell and islet cotransplantation: safety and efficacy. Stem Cells Transl Med. (2018) 7:11–9. doi: 10.1002/sctm.17-0139 PMC5746145 29159905

[91] Charbonnier LM Cui Y Stephen-Victor E Harb H Lopez D Bleesing JJ. Functional reprogramming of regulatory T cells in the absence of Foxp3. Nat Immunol. (2019) 20:1208–19. doi: 10.1038/s41590-019-0442-x PMC6707855 31384057

[92] Martin-Moreno PL Tripathi S Chandraker A. Regulatory T cells and kidney transplantation. Clin J Am Soc Nephrol. (2018) 13:1760–4. doi: 10.2215/CJN.01750218 PMC6237070 29789350

[93] Romano M Fanelli G Albany CJ Giganti G Lombardi G. Past, present, and future of regulatory T cell therapy in transplantation and autoimmunity. Front Immunol. (2019) 10:43. doi: 10.3389/fimmu.2019.00043 30804926 PMC6371029

[94] Sakaguchi S. Naturally arising Foxp3-expressing CD25+CD4+ regulatory T cells in immunological tolerance to self and non-self. Nat Immunol. (2005) 6:345–52. doi: 10.1038/ni1178 15785760

[95] Yoon IH Chung H Kim HJ Nam HY Shin JS Kim YH. Peri-graft porcine-specific CD4+ FoxP3+ regulatory T cells by CD40-CD154 blockade prevented the rejection of porcine islet graft in diabetic mice. Xenotransplantation. (2019) 26:e12533. doi: 10.1111/xen.12533 31111577

[96] Gregori S Casorati M Amuchastegui S Smiroldo S Davalli AM Adorini L. Regulatory T cells induced by 1 alpha,25-dihydroxyvitamin D3 and mycophenolate mofetil treatment mediate transplantation tolerance. J Immunol. (2001) 167:1945–53. doi: 10.4049/jimmunol.167.4.1945 11489974

[97] Gagliani N Jofra T Valle A Stabilini A Morsiani C Gregori S. Transplant tolerance to pancreatic islets is initiated in the graft and sustained in the spleen. Am J Transplant. (2013) 13:1963–75. doi: 10.1111/ajt.12333 PMC3869180 23834659

[98] Grinberg-Bleyer Y Baeyens A You S Elhage R Fourcade G Gregoire S. IL-2 reverses established type 1 diabetes in NOD mice by a local effect on pancreatic regulatory T cells. J Exp Med. (2010) 207:1871–8. doi: 10.1084/jem.20100209 PMC2931175 20679400

[99] Tang Q Henriksen KJ Bi M Finger EB Szot G Ye J. *In vitro*-expanded antigen-specific regulatory T cells suppress autoimmune diabetes. J Exp Med. (2004) 199:1455–65. doi: 10.1084/jem.20040139 PMC2211775 15184499

[100] Gagliani N Jofra T Stabilini A Valle A Atkinson M Roncarolo MG. Antigen-specific dependence of Tr1-cell therapy in preclinical models of islet transplant. Diabetes. (2010) 59:433–9. doi: 10.2337/db09-1168 PMC2809952 19934002

[101] Lee K Nguyen V Lee KM Kang SM Tang Q. Attenuation of donor-reactive T cells allows effective control of allograft rejection using regulatory T cell therapy. Am J Transplant. (2014) 14:27–38. doi: 10.1111/ajt.12509 24354870 PMC5262439

[102] Pierini A Iliopoulou BP Peiris H Pérez-Cruz M Baker J Hsu K. T cells expressing chimeric antigen receptor promote immune tolerance. JCI Insight. (2017) 2:e92865. doi: 10.1172/jci.insight.92865 29046484 PMC5846896

[103] Chang TM. SEMIPERMEABLE MICROCAPSULES. Science. (1964) 146:524–5. doi: 10.1126/science.146.3643.524 14190240

[104] Olabisi RM. Cell microencapsulation with synthetic polymers. J BioMed Mater Res A. (2015) 103:846–59. doi: 10.1002/jbm.a.35205 PMC4309473 24771675

[105] Mallett AG Korbutt GS. Alginate modification improves long-term survival and function of transplanted encapsulated islets. Tissue Eng Part A. (2009) 15:1301–9. doi: 10.1089/ten.tea.2008.0118 18950258

[106] Chen T Yuan J Duncanson S Hibert ML Kodish BC Mylavaganam G. Alginate encapsulant incorporating CXCL12 supports long-term allo- and xenoislet transplantation without systemic immune suppression. Am J Transplant. (2015) 15:618–27. doi: 10.1111/ajt.13049 25693473

[107] Alagpulinsa DA Cao JJL Driscoll RK Sîrbulescu RF Penson MFE Sremac M. Alginate-microencapsulation of human stem cell-derived β cells with CXCL12 prolongs their survival and function in immunocompetent mice without systemic immunosuppression. Am J Transplant. (2019) 19:1930–40. doi: 10.1111/ajt.15308 30748094

[108] Enck K Tamburrini R Deborah C Gazia C Jost A Khalil F. Effect of alginate matrix engineered to mimic the pancreatic microenvironment on encapsulated islet function. Biotechnol Bioeng. (2021) 118:1177–85. doi: 10.1002/bit.27641 PMC8887826 33270214

[109] Krishtul S Skitel Moshe M Kovrigina I Baruch L Machluf M. ECM-based bioactive microencapsulation significantly improves islet function and graft performance. Acta Biomater. (2023) 171:249–60. doi: 10.1016/j.actbio.2023.09.009 37708927

[110] Goswami D Domingo-Lopez DA Ward NA Millman JR Duffy GP Dolan EB. Design considerations for macroencapsulation devices for stem cell derived islets for the treatment of type 1 diabetes. Adv Sci (Weinh). (2021) 8:e2100820. doi: 10.1002/advs.202100820 34155834 PMC8373111

[111] Kasoju N Pátíková A Wawrzynska E Vojtíšková A Sedlačík T Kumorek M. Bioengineering a pre-vascularized pouch for subsequent islet transplantation using VEGF-loaded polylactide capsules. Biomater Sci. (2020) 8:631–47. doi: 10.1039/C9BM01280J 31729495

[112] Wang LH Marfil-Garza BA Ernst AU Pawlick RL Pepper AR Okada K. Inflammation-induced subcutaneous neovascularization for the long-term survival of encapsulated islets without immunosuppression. Nat BioMed Eng. (2023), 10.1038/s41551-023-01145-8. doi: 10.1038/s41551-023-01145-8 38052996

[113] Wang X Maxwell KG Wang K Bowers DT Flanders JA Liu W. A nanofibrous encapsulation device for safe delivery of insulin-producing cells to treat type 1 diabetes. Sci Transl Med. (2021) 13:eabb4601. doi: 10.1126/scitranslmed.abb4601 34078744 PMC8563008

[114] Liu W Flanders JA Wang LH Liu Q Bowers DT Wang K. A safe, fibrosis-mitigating, and scalable encapsulation device supports long-term function of insulin-producing cells. Small. (2022) 18:e2104899. doi: 10.1002/smll.202104899 34897997 PMC8881301

[115] Li H He W Feng Q Chen J Xu X Lv C. Engineering superstable islets-laden chitosan microgels with carboxymethyl cellulose coating for long-term blood glucose regulation *in vivo* . Carbohydr Polym. (2024) 323:121425. doi: 10.1016/j.carbpol.2023.121425 37940297

[116] Li H Shang Y Feng Q Liu Y Chen J Dong H. A novel bioartificial pancreas fabricated via islets microencapsulation in anti-adhesive core-shell microgels and macroencapsulation in a hydrogel scaffold prevascularized *in vivo* . Bioact Mater. (2023) 27:362–76. doi: 10.1016/j.bioactmat.2023.04.011 PMC10172916 37180642

[117] Yang K O’Cearbhaill ED Liu SS Zhou A Chitnis GD Hamilos AE. A therapeutic convection-enhanced macroencapsulation device for enhancing β cell viability and insulin secretion. Proc Natl Acad Sci U S A. (2021) 118:e2101258118. doi: 10.1073/pnas.2101258118 34504013 PMC8449352

[118] Shaheen R Gurlin RE Gologorsky R Blaha C Munnangi P Santandreu A. Superporous agarose scaffolds for encapsulation of adult human islets and human stem-cell-derived β cells for intravascular bioartificial pancreas applications. J BioMed Mater Res A. (2021) 109:2438–48. doi: 10.1002/jbm.a.37236 PMC13653688 34196100

[119] Harrington S Karanu F Ramachandran K Williams SJ Stehno-Bittel L. PEGDA microencapsulated allogeneic islets reverse canine diabetes without immunosuppression. PloS One. (2022) 17:e0267814. doi: 10.1371/journal.pone.0267814 35613086 PMC9132281

[120] Stock AA Manzoli V De Toni T Abreu MM Poh YC Ye L. Conformal coating of stem cell-derived islets for β Cell replacement in type 1 diabetes. Stem Cell Rep. (2020) 14:91–104. doi: 10.1016/j.stemcr.2019.11.004 PMC6962554 31839542

[121] Schmidt C. Pancreatic islets find a new transplant home in the omentum. Nat Biotechnol. (2017) 35:8. doi: 10.1038/nbt0117-8 28072777

[122] Johansson H Goto M Dufrane D Siegbahn A Elgue G Gianello P. Low molecular weight dextran sulfate: a strong candidate drug to block IBMIR in clinical islet transplantation. Am J Transplant. (2006) 6:305–12. doi: 10.1111/j.1600-6143.2005.01186.x 16426314

[123] Schaschkow A Mura C Pinget M Bouzakri K Maillard E. Intra-Omental Islet Transplantation Using h-Omental Matrix Islet filliNG (hOMING). J Vis Exp. (2019) 145):10. doi: 10.3791/58898 30933067

[124] Saudek F Hladiková Z Hagerf B Nemetova L Girman P Kriz J. Transplantation of pancreatic islets into the omentum using a biocompatible plasma-thrombin gel: first experience at the institute for clinical and experimental medicine in prague. Transplant Proc. (2022) 54:806–10. doi: 10.1016/j.transproceed.2021.11.037 35227510

[125] Hladíková Z Voglová B Pátíková A Berková Z Kříž J Vojtíšková A. Bioluminescence imaging *in vivo* confirms the viability of pancreatic islets transplanted into the greater omentum. Mol Imaging Biol. (2021) 23:639–49. doi: 10.1007/s11307-021-01588-y 33599904

[126] Rafael E Tibell A Rydén M Lundgren T Sävendahl L Borgström B. Intramuscular autotransplantation of pancreatic islets in a 7-year-old child: a 2-year follow-up. Am J Transplant. (2008) 8:458–62. doi: 10.1111/j.1600-6143.2007.02060.x 18093267

[127] Park JL Kim T Kim BK. Suitability of denervated muscle flaps as recipient sites for pancreatic islet cell transplantation. Arch Plast Surg. (2021) 48:133–43. doi: 10.5999/aps.2020.01865 PMC7861985 33503758

[128] Rojas-Canales D Walters SN Penko D Cultrone D Bailey J Chtanova T. Intracutaneous transplantation of islets within a biodegradable temporizing matrix as an alternative site for islet transplantation. Diabetes. (2023) 72:758–68. doi: 10.2337/db21-0841 PMC10202765 36929171

[129] Kinney SM Ortaleza K Vlahos AE Sefton MV. Degradable methacrylic acid-based synthetic hydrogel for subcutaneous islet transplantation. Biomaterials. (2022) 281:121342. doi: 10.1016/j.biomaterials.2021.121342 34995903

[130] Perez VL Caicedo A Berman DM Arrieta E Abdulreda MH Rodriguez-Diaz R. The anterior chamber of the eye as a clinical transplantation site for the treatment of diabetes: a study in a baboon model of diabetes. Diabetologia. (2011) 54:1121–6. doi: 10.1007/s00125-011-2091-y PMC3247302 21360190

[131] Litbarg NO Gudehithlu KP Sethupathi P Arruda JA Dunea G Singh AK. Activated omentum becomes rich in factors that promote healing and tissue regeneration. Cell Tissue Res. (2007) 328:487–97. doi: 10.1007/s00441-006-0356-4 17468892

[132] Damyar K Farahmand V Whaley D Alexander M Lakey JRT. An overview of current advancements in pancreatic islet transplantation into the omentum. Islets. (2021) 13:115–20. doi: 10.1080/19382014.2021.1954459 PMC8528405 34402725

[133] Baidal DA Ricordi C Berman DM Alvarez A Padilla N Ciancio G. Bioengineering of an intraabdominal endocrine pancreas. N Engl J Med. (2017) 376:1887–9. doi: 10.1056/NEJMc1613959 PMC5572072 28489987

[134] Yu M Agarwal D Korutla L May CL Wang W Griffith NN. Islet transplantation in the subcutaneous space achieves long-term euglycaemia in preclinical models of type 1 diabetes. Nat Metab. (2020) 2:1013–20. doi: 10.1038/s42255-020-0269-7 PMC7572844 32895576

[135] Lei J Zhang A Deng H Yang Z Peters CW Lee KM. Intrapleural transplantation of allogeneic pancreatic islets achieves glycemic control in a diabetic non-human primate. Am J Transplant. (2022) 22:966–72. doi: 10.1111/ajt.16875 PMC8897220 34704352

[136] Zhang M Du H Guan Y Liu J Wang S Li H. Study on the effect of PDA-PLGA scaffold loaded with islet cells for skeletal muscle transplantation in the treatment of diabetes. Front Bioeng Biotechnol. (2022) 10:927348. doi: 10.3389/fbioe.2022.927348 35845408 PMC9280155

[137] Ilegems E Berggren PO. The eye as a transplantation site to monitor pancreatic islet cell plasticity. Front Endocrinol (Lausanne). (2021) 12:652853. doi: 10.3389/fendo.2021.652853 33967961 PMC8104082

[138] Cayabyab F Nih LR Yoshihara E. Advances in pancreatic islet transplantation sites for the treatment of diabetes. Front Endocrinol (Lausanne). (2021) 12:732431. doi: 10.3389/fendo.2021.732431 34589059 PMC8473744

[139] Wagner LE Melnyk O Duffett BE Linnemann AK. Mouse models and human islet transplantation sites for intravital imaging. Front Endocrinol (Lausanne). (2022) 13:992540. doi: 10.3389/fendo.2022.992540 36277698 PMC9579277

[140] Li F Lv Y Li X Yang Z Guo T Zhang J. Comparative study of two different islet transplantation sites in mice: hepatic sinus tract vs splenic parenchyma. Cell Transplant. (2020) 29:963689720943576. doi: 10.1177/0963689720943576 32731817 PMC7563812

[141] Szempruch KR Banerjee O McCall RC Desai CS. Use of anti-inflammatory agents in clinical islet cell transplants: A qualitative systematic analysis. Islets. (2019) 11:65–75. doi: 10.1080/19382014.2019.1601543 31149871 PMC6548473

[142] Westwell-Roper C Dai DL Soukhatcheva G Potter KJ van Rooijen N Ehses JA. IL-1 blockade attenuates islet amyloid polypeptide-induced proinflammatory cytokine release and pancreatic islet graft dysfunction. J Immunol. (2011) 187:2755–65. doi: 10.4049/jimmunol.1002854 21813778

[143] McCall M Pawlick R Kin T Shapiro AM. Anakinra potentiates the protective effects of etanercept in transplantation of marginal mass human islets in immunodeficient mice. Am J Transplant. (2012) 12:322–9. doi: 10.1111/j.1600-6143.2011.03796.x 22053751

[144] Abdel-Karim TR Hodges JS Herold KC Pruett TL Ramanathan KV Hering BJ. Peri-transplant inflammation and long-term diabetes outcomes were not impacted by either etanercept or alpha-1-antitrypsin treatment in islet autotransplant recipients. Transpl Int. (2024) 37:12320. doi: 10.3389/ti.2024.12320 38357216 PMC10864605

[145] Shahaf G Moser H Ozeri E Mizrahi M Abecassis A Lewis EC. α-1-antitrypsin gene delivery reduces inflammation, increases T-regulatory cell population size and prevents islet allograft rejection. Mol Med. (2011) 17:1000–11. doi: 10.2119/molmed.2011.00145 PMC3188864 21670848

[146] Wang J Sun Z Gou W Adams DB Cui W Morgan KA. α-1 antitrypsin enhances islet engraftment by suppression of instant blood-mediated inflammatory reaction. Diabetes. (2017) 66:970–80. doi: 10.2337/db16-1036 PMC5360304 28069642

[147] Baysoy A Bai Z Satija R Fan R. The technological landscape and applications of single-cell multi-omics. Nat Rev Mol Cell Biol. (2023) 24:695–713. doi: 10.1038/s41580-023-00615-w 37280296 PMC10242609

[148] Mohiuddin MM Singh AK Scobie L Goerlich CE Grazioli A Saharia K. Graft dysfunction in compassionate use of genetically engineered pig-to-human cardiac xenotransplantation: a case report. Lancet. (2023) 402:397–410. doi: 10.1016/S0140-6736(23)00775-4 37393920 PMC10552929

